# Acoustic focusing of beads and cells in hydrogel droplets

**DOI:** 10.1038/s41598-021-86985-7

**Published:** 2021-04-05

**Authors:** Anna Fornell, Hannah Pohlit, Qian Shi, Maria Tenje

**Affiliations:** 1 Department of Materials Science and Engineering, Science for Life Laboratory, Uppsala University, 75121 Uppsala, Sweden; 2 MAXIV Laboratory, Lund University, 22484 Lund, Sweden

**Keywords:** Biophysics, Biomedical engineering

## Abstract

The generation of hydrogel droplets using droplet microfluidics has emerged as a powerful tool with many applications in biology and medicine. Here, a microfluidic system to control the position of particles (beads or astrocyte cells) in hydrogel droplets using bulk acoustic standing waves is presented. The chip consisted of a droplet generator and a 380 µm wide acoustic focusing channel. Droplets comprising hydrogel precursor solution (polyethylene glycol tetraacrylate or a combination of polyethylene glycol tetraacrylate and gelatine methacrylate), photoinitiator and particles were generated. The droplets passed along the acoustic focusing channel where a half wavelength acoustic standing wave field was generated, and the particles were focused to the centre line of the droplets (i.e. the pressure nodal line) by the acoustic force. The droplets were cross-linked by exposure to UV-light, freezing the particles in their positions. With the acoustics applied, 89 ± 19% of the particles (polystyrene beads, 10 µm diameter) were positioned in an area ± 10% from the centre line. As proof-of-principle for biological particles, astrocytes were focused in hydrogel droplets using the same principle. The viability of the astrocytes after 7 days in culture was 72 ± 22% when exposed to the acoustic focusing compared with 70 ± 19% for samples not exposed to the acoustic focusing. This technology provides a platform to control the spatial position of bioparticles in hydrogel droplets, and opens up for the generation of more complex biological hydrogel structures.

## Introduction

Hydrogels are a class of materials that are composed of a network of polymer chains that contain a large amount of water. They are interesting for a variety of biological applications such as biosensors, cell culturing scaffolds and drug delivery systems^1^. They can be formed into different shapes, and one promising approach is to use droplet microfluidics to fabricate micrometre scale hydrogel droplets containing cells or other particles^2–5^. There are different terms to define these cross-linked hydrogel droplets: beads, droplets, particles, microcapsules and microgels; in this work we use the term “hydrogel droplets”.

In droplet microfluidic systems two immiscible fluids, commonly water and oil, meet in a cross-section resulting in the breakup of monodisperse water droplets surrounded by oil^6^. By injecting a hydrogel precursor solution instead of water, droplets consisting of hydrogel precursor solution are generated and these droplets can then be cross-linked. Cross-linking can be induced using various means such as chemicals, a temperature change or exposure to UV-light.

Compared with water-in-oil droplets, hydrogel droplets are easier to handle in later analysis steps as they are stable and do not risk coalescing. Moreover, hydrogel droplets can provide a 3D scaffold structure for cell culture^7^ and they are also interesting for drug delivery^8,9^ and tissue engineering applications^10^. After cross-linking, the hydrogel droplets are typically washed to remove the oil and surfactant, and suspended in an aqueous solution such as a buffer solution or cell medium. Suspending the hydrogel droplets in an aqueous solution allows for exchange of water-soluble molecules, e.g. nutrients and chemicals between the hydrogel material and the surrounding solution.

Hydrogel droplets can be produced in various materials and material combinations. The composition of the hydrogel material should be selected depending on the intended application. Previously, hydrogel droplets have for example been produced in hydrogels from natural-derived polymers such as alginate^11^, agarose^12^ and hyaluronic acid^13^ as well as hydrogels from synthetic polymers such as polyethylene glycol (PEG)^14^. In this work we have selected to generate PEG droplets as PEG is a cheap, inert, highly water-soluble and well-characterised material^15^, and the precursor solution has low viscosity compared with many other hydrogel precursor solutions^16^. The viscosity of the dispersed phase (i.e. the hydrogel precursor solution) determines the droplet generation regime and it also affects the size of the droplets^17^, and generally a low viscosity is favourable for stable droplet formation. Having a low viscosity is also advantageous for the acoustic focusing as it decreases the acoustic focusing time. For biological applications it is important to consider the biocompatibility of the gel composition. One commonly occurring problem with synthetic hydrogels is that they are not enzymatically degradable and do not support cell adhesion^5^. To improve the biocompatibility of the PEG gels, we therefore added gelatine methacrylate (GelMA) to the PEG solution in the cell experiments. Gelatine is a suitable material for 3D cell cultures because it provides enzymatically degradable sites as well as cell attachment points essential for cell adherence and proliferation^18^. However, our initial experiments showed that pure GelMA is not suitable for acoustic focusing due to the high viscosity.

There are several techniques that have been applied to position beads and cells in hydrogels including acoustic forces^19–21^, dielectrophoresis^22^ and bioprinting^23^. Most research has focused on manipulation of particles in bulk hydrogels and fibres, and there are only a few reports on manipulation of particles in hydrogel droplets. Kamperman et al. observed centring of cells encapsulated in hydrogel droplets by delayed cross-linking of the hydrogel. The physics accounting for the effect is not completely known, but it is expected to be related to the droplet movement along the channel^24^. Similarly, Lienemann et al*.* report that shaking the droplets during gelation using an orbital shaker results in centring of the encapsulated cells^25^. The applications of controlled particle positioning include for example preventing “cell escape”^7,24^. It is known that cells that are positioned close to the border of the hydrogel droplets frequently escape from the droplets which limits long term cell culture, and by centring the cells this can avoided^24^. Other applications include 3D cell cultures in a droplet microfluidic platform^26^. The technology also holds potential to be integrated into multilayer droplet hydrogel structures. In this paper we report for the first time, an on-chip and direct method to control the alignment of particles in hydrogel droplets using bulk acoustic standing waves.

Acoustic forces have been used in a wide range of applications to enrich and sort particles^27^, and lately for manipulating encapsulated particles in water-in-oil droplets^28–30^. Acoustic particle manipulation has many advantages such as being actively controlled, label-free, operated in non-contact mode and having high biocompatibility^31^. Moreover, the method is independent of the particle charge and the ionic strength of the fluid. However, to obtain strong acoustic focusing in two-phase systems, the continuous phase should have similar acoustic properties as the dispersed phase^32^. Typically, in droplet microfluidics the continuous phase is a fluorocarbon oil, but fluorocarbon oils have very different acoustic properties compared to the dispersed phase (often an aqueous solution for biological applications). Therefore, for intra-droplet acoustic particle focusing fluorocarbon oils are not suitable to use. In this work we selected to use mineral oil as the continuous phase, since it has similar acoustic properties^33^ as the dispersed phase. Mineral oil has previously been used in several studies to generate droplets with cells encapsulated^34–36^.

In particle focusing experiments using bulk acoustic standing waves, acoustic resonance is set in the microfluidic channel by matching the frequency of the sound to the channel dimensions. Particles in an acoustic standing wave field experience an acoustic force, the primary acoustic radiation force, which moves the particles to the pressure nodal line. At half wavelength lateral resonance, the pressure nodal line is located along the centre line of the channel, thus particles such as plastic beads or cells are focused along the centre line of the channel. The primary acoustic radiation force acting on a particle in a one-dimensional half wavelength acoustic standing wave field is given by,

1 $${F}_{\mathrm{y}}^{\mathrm{rad}}=4\mathrm{\pi \Phi }(\stackrel{\sim }{\kappa ,}\stackrel{\sim }{\rho })k{a}^{3}{E}_{\mathrm{ac}}\mathrm{sin}(2ky)$$

2 $$\Phi \left(\stackrel{\sim }{\kappa ,}\stackrel{\sim }{\rho }\right)=\frac{1}{3}\left[\frac{5\stackrel{\sim }{\rho }-2}{2\stackrel{\sim }{\rho }+1}-\stackrel{\sim }{\kappa }\right]$$

where λ is the wavelength of the sound, $\Phi$ is the acoustic contrast factor, $\stackrel{\sim }{\kappa }$ is the compressibility ratio between the particle and the fluid, $\stackrel{\sim }{\rho }$ is the density ratio between the particle and the fluid, $k$ is the wavenumber ($k=2\uppi /\lambda )$, $a$ is the particle radius, ${E}_{\mathrm{ac}}$ is the acoustic energy density and $y$ is the distance from the channel wall^37^. The velocity $(v$) of a particle moving in a fluid is inversely proportional to the viscosity of the fluid and is given by,

3 $$v(y)=\frac{2\Phi \left(\stackrel{\sim }{\kappa },\stackrel{\sim }{\rho }\right)k{a}^{2}{E}_{\mathrm{ac}}\mathrm{sin}(2ky)}{3\eta }$$

where $\eta$ is the viscosity of the fluid^37^. Hydrogel precursor solutions are generally more viscous than water^16^, thus it is important to have a strong acoustic force to be able to focus the encapsulated particles.

### Operating principle

**Figure 1:**
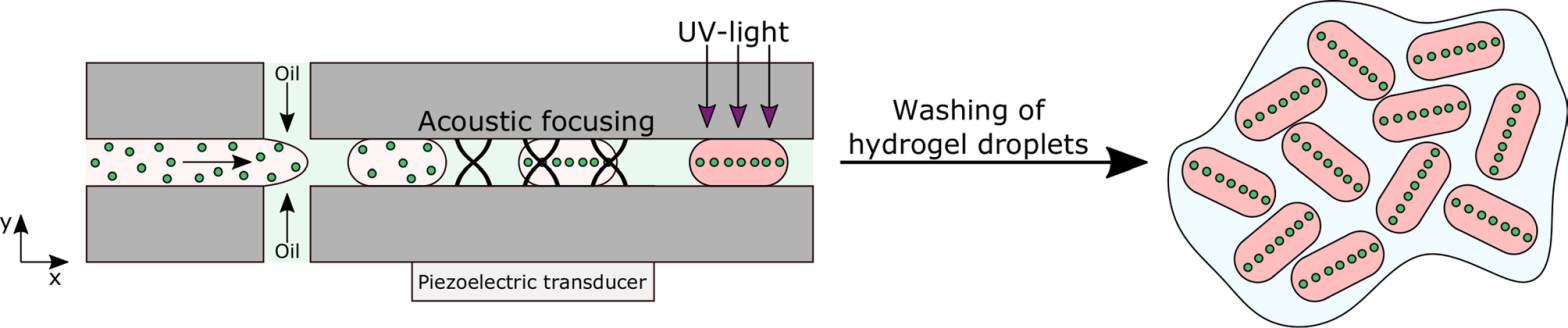
Droplets composed of hydrogel precursor solution (PEG or a combination of PEG and GelMA), photoinitiator and particles (beads or cells) are generated. The encapsulated particles are focused along the centre line of the droplets by the primary acoustic radiation force and cross-linked by exposure to UV-light. The hydrogel droplets are collected and washed to remove the oil and surfactant.

The concept of acoustic particle focusing in hydrogel droplets is shown schematically in Fig. 1. Droplets composed of hydrogel precursor solution (PEG or a combination of PEG and GelMA), photoinitiator and particles (beads or cells) are generated in a flow focusing design. The droplets are passing downstream along the acoustic focusing channel. In the acoustic focusing channel a one-dimensional half wavelength acoustic standing wave field is generated by a piezoelectric transducer. This causes the encapsulated particles to be focused by the primary acoustic radiation force (Eq. 1) along the centre line of the droplets (i.e. the pressure nodal line). Since the droplets are not cross-linked yet, the particles are free to be moved inside the droplets. Further downstream the channel, the droplets are cross-linked by exposure to UV-light which freezes the particles in their positions. We selected to use UV-light induced cross-linking, because it is important that the cross-linking occurs on the chip so the particles are still focused by the acoustics. The hydrogel droplets are then collected, washed to remove the oil and surfactant, and suspended in an aqueous solution.

## Results and discussion

### Acoustic focusing of beads in hydrogel droplets

PEG droplets containing polystyrene beads (0.5 µm red beads and 10 µm green beads) were generated in the microfluidic chip. The aim was to focus the 10 µm beads, while the 0.5 µm beads were added for visualisation of the droplets since the interface of the hydrogel droplets can be hard to distinguish when suspended in an aqueous solution. A water-soluble fluorescent dye is not suitable to use as it can diffuse out of the droplets into the aqueous solution.

**Figure 2:**
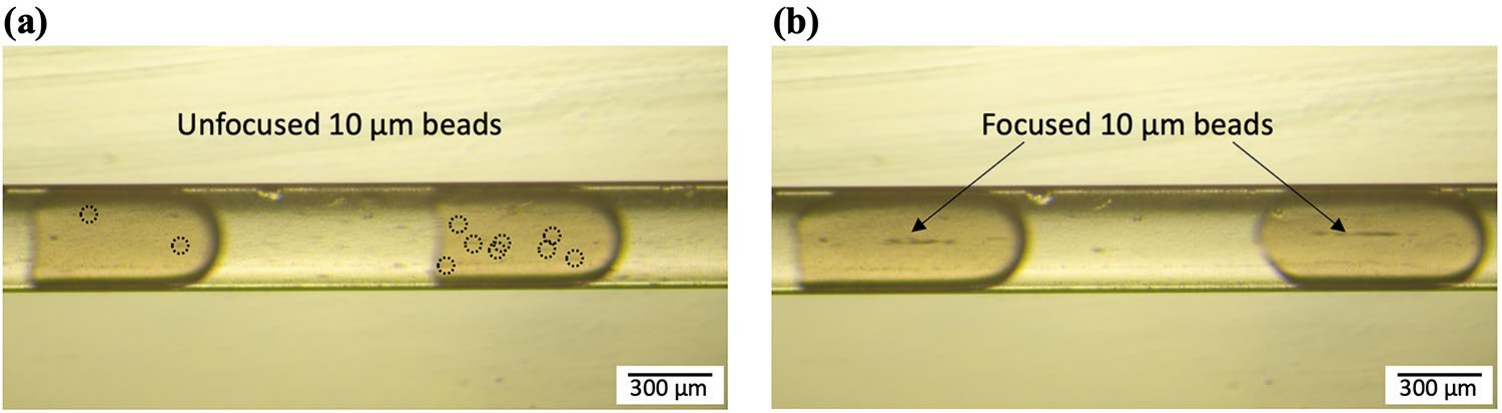
PEG droplets in the acoustic focusing channel before cross-linking. **(a)** Without the acoustics applied the 10 µm beads are randomly positioned in the droplets. The dashed circles indicate the beads. **(b)** With the acoustics applied the 10 µm beads are focused along the centre line of the droplets. The flow direction is towards the right in the photos.

After droplet generation the droplets passed along the acoustic focusing channel. The piezoelectric transducer was actuated, and a one-dimensional half wavelength acoustic standing wave field was generated between the channel walls in the acoustic focusing channel. The frequency of the applied signal was matched to the channel width, and the best acoustic focusing was observed at 1.88 MHz. In Fig. 2 photos of droplets passing the acoustic focusing channel before cross-linking are shown. Without the acoustics applied, the 10 µm beads were distributed in the entire droplets (Fig. 2a), but when the acoustics was applied the 10 µm beads were focused along the centre line of the droplets (Fig. 2b).

In this work acoustic resonance was only set in the y-direction, thus the particles were only focused in that direction. However, it would be possible to focus the particles also in the z-direction^38^ by using either a square microfluidic channel or by using a second transducer matched to the channel height.

Compared with acoustic focusing in water-in-oil droplets, acoustic focusing in hydrogel droplets is more challenging since hydrogel precursor solutions are more viscous than water. In this experiment 3% PEG was used. The viscosity of 3% PEG is 1.2 times higher than PBS (phosphate buffered saline), 5.6 mPas compared with 4.6 mPas at room temperature. As seen in Fig. 2b it was possible to focus the 10 µm beads in 3% PEG droplets. The average flow velocity in the main channel was low (1.1 mm/s) to ensure sufficient time for the acoustic forces to interact with the encapsulated beads. Initial experiments were performed to focus beads in droplets composed of 8% GelMA (which is a typically concentration) instead of 3% PEG. It was found to be harder to focus the beads in 8% GelMA compared with 3% PEG (data not shown) and a higher actuation voltage was required. This is related to the much higher viscosity of 8% GelMA (3,941 mPas) compared with 3% PEG (5.6 mPas) at room temperature. In addition to impairing the acoustic focusing, the high viscosity also affects the droplet generation. Therefore, we selected to work with 3% PEG. However, in the cell experiments 1% GelMA was added to the 3% PEG solution to improve the cell viability (see section “Acoustic focusing of astrocytes in hydrogel droplets”). The viscosity of the combination of 3% PEG and 1% GelMA is 7.7 mPas at room temperature.

After acoustic focusing the droplets passed downstream the channel, and at the end of the channel the droplets were cross-linked by exposure to UV-light. In addition to ensuring sufficient time for the acoustic focusing, the low flow velocity also ensured that there was sufficient time for the UV-light exposure. The measured stiffness of 3% PEG gel after cross-linking was 723 Pa.

**Figure 3:**
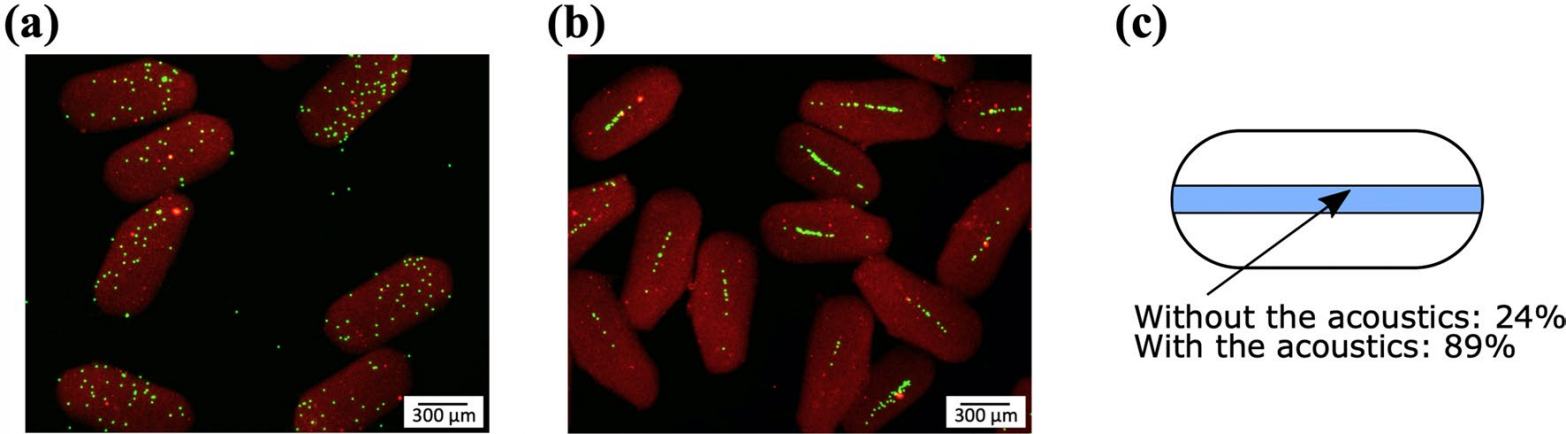
Hydrogel droplets with encapsulated beads after cross-linking and washing **(a)** Without the acoustics applied the 10 µm green beads are randomly positioned in the droplets. **(b) **With the acoustics applied the 10 µm green beads are focused along the centre line of the droplets. **(c) **The amount of 10 µm green fluorescent beads in the region of interest (ROI, marked in blue in the image) without and with the acoustics applied.

The droplets were collected by connecting a 10 cm long tubing from the outlet of the chip to an Eppendorf tube. After cross-linking the droplets were stable and could be easily handled without risk of coalescing or being damaged. The droplets were washed with PBS to remove the oil and surfactant. Mineral oil has lower density (0.86 g/cm^3^) than both hydrogel droplets and PBS, thus the hydrogel droplets sedimented and could be collected in the PBS. In Fig. 3 fluorescent images of the cross-linked and washed hydrogel droplets are shown. Since the droplets were cross-linked the encapsulated beads were frozen in their positions. Without the acoustics applied, the 10 µm beads were randomly distributed in the droplets (Fig. 3a) while with the acoustics applied the 10 µm beads were focused along the centre line of the droplets (Fig. 3b). With the acoustics applied 89 ± 19% (s.d.) of the 10 µm beads were positioned in an area ± 10% from the centre line of the droplets compared with 24 ± 12% (s.d.) without the acoustics applied (Fig. 3c).

The hydrogel precursor solution contained 0.5 µm red beads to facilitate visualisation of the hydrogel droplets after washing. As seen in Fig. 3b the 0.5 µm beads were not focused by the acoustics. The acoustic radiation force is strongly dependent on the size of the beads (Eq. 1), and the force was not strong enough to focus the 0.5 µm beads.

### Acoustic focusing of beads in different droplet lengths

**Figure 4:**
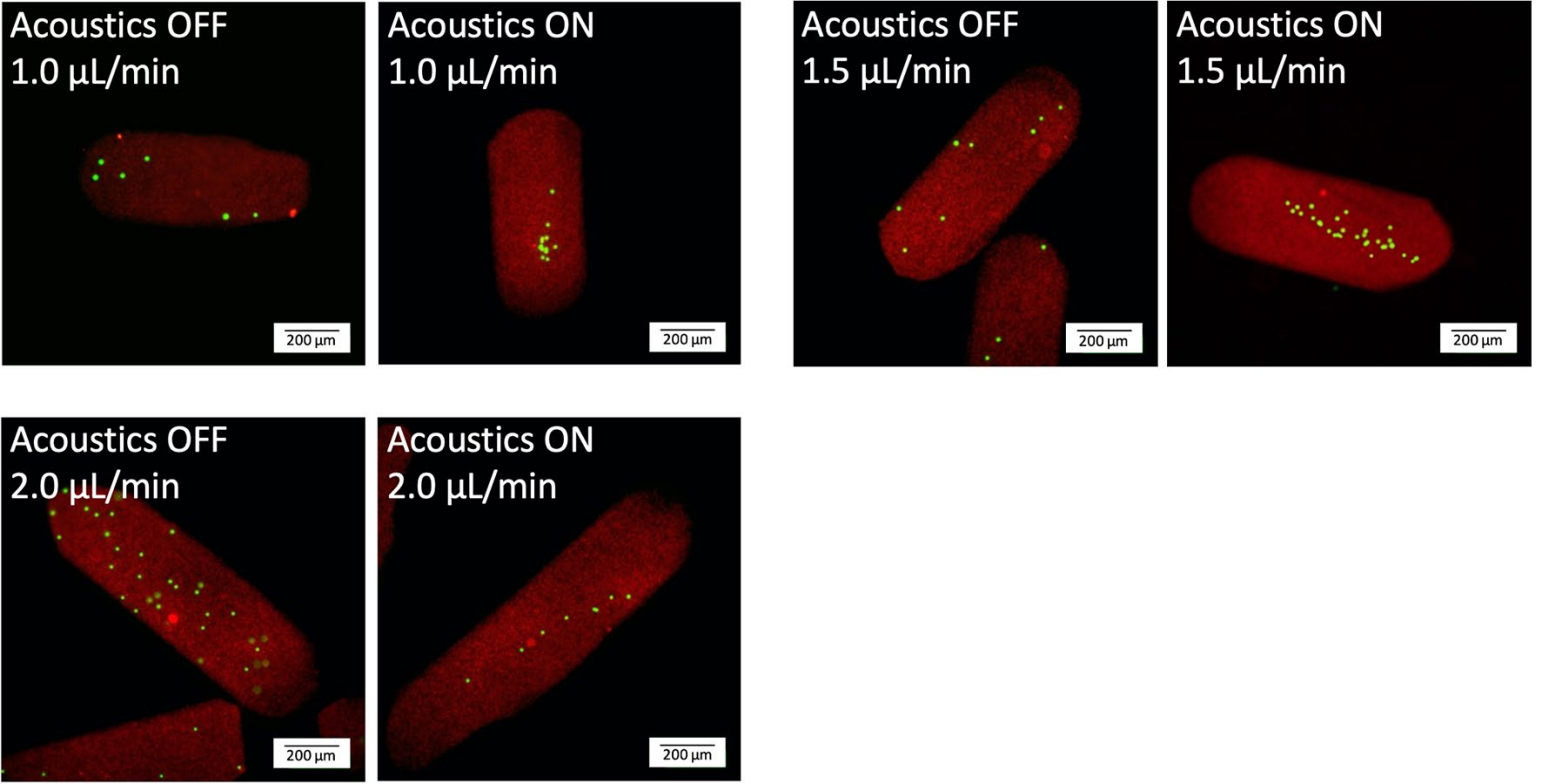
Acoustic focusing in different droplet lengths. The photos show the hydrogel droplets after cross-linking and washing. The flow rate of the oil was 1.5 µL/min while the flow rate of the hydrogel precursor solution was varied between 1.0 and 2.0 µL/min as indicated in the photos.

The size of the droplets depends on the channel dimensions and the flow rates of the continuous and dispersed phases^17^. In this work the channel width was designed to be 380 µm wide which supports $\lambda$/2-wavelength resonance when actuated with a 2 MHz transducer. Experiments to generate droplets of different lengths were performed. In these experiments the flow rate of the oil was kept constant (1.5 µL/min) while the flow rate of the hydrogel precursor solution was varied between 1.0 and 2.0 µL/min. A low flow rate of the hydrogel precursor solution resulted in short droplets whereas a high flow rate resulted in longer droplets (Fig. 4). The experiments show that it is possible to focus beads in droplets of different lengths. In the experiments it was observed that if the droplets were very long, there was an increased risk that the channel was clogged during cross-linking and the flow was stopped. It was also observed that it was more difficult to focus particles in short droplets compared with longer droplets (data not shown). This is in line with what has been observed for water-in-oil droplets^39^. However, more studies on the physics involved in acoustic particle manipulation in droplet microfluidic systems are required to fully understand the interplay of the droplet length, the fluid compositions, the internal motions, sedimentation etc. on the acoustic focusing quality.

### Acoustic focusing of astrocytes in hydrogel droplets

As proof-of-principle of the acoustic focusing technology, focusing of cells inside hydrogel droplets was investigated. In this experiment droplets consisting of a combination of 3% PEG and 1% GelMA were generated and astrocytes were encapsulated. GelMA was added to the PEG solution because it provides enzymatically degradable sites and cell attachment points^18^. As described above, pure GelMA (8%) is not suitable for acoustic focusing as the viscosity is too high which makes the acoustic focusing difficult.

In the acoustic focusing experiments droplets containing cells passed along the acoustic focusing channel. The acoustic focusing worked with the combination of 3% PEG and 1% GelMA (viscosity of 7.7 mPas at room temperature). Without the acoustics applied the cells were positioned in the entire droplets, while with the acoustics applied the cells were focused inside the droplets. In the experiments it was observed that it is harder to focus the cells than the beads. There are two possible reasons. First, the viscosity of the gel combination used in the cell experiments is higher than for the bead experiments. Secondly, the acoustic contrast factor is generally lower for cells compared with polystyrene beads, thus it is expected to be more difficult to focus cells compared with polystyrene beads. To improve the acoustic focusing of the cells a higher voltage could be used, however by increasing the voltage the temperature in the chip increases which can be harmful for the cells^40^.

After acoustic focusing, the droplets were cross-linked by exposure to UV-light and washed to remove the oil and surfactant. The measured stiffness of the combination of 3% PEG and 1% GelMA gel after cross-linking was 1,439 Pa. The chosen hydrogel composition was chosen to be biological relevant for cells where it for example matches the stiffness of brain tissue (685–1875 Pa)^41^ which is the natural environment of astrocytes.

**Figure 5:**
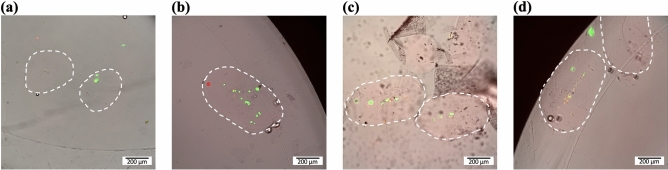
Hydrogel droplets with encapsulated astrocytes after cross-linking and washing **(a)** Without the acoustics applied the cells are randomly positioned in the hydrogel droplets. The photo is taken after 7 days of cell culture. **(b–d)** With the acoustics applied the cells are focused along the centre line of the hydrogel droplets. **(b)** shows the cells after 1 day of culture, **(c)** shows the cells after 3 days of culture and **(d)** shows the cells after 7 days of culture. The dashed lines indicate the border of the hydrogel droplets. Living cells stained with calcein appear green whereas dead cells stained with propidium iodide appear red.

The cross-linked droplets were incubated in cell media at 37 ºC for 1, 3 or 7 days, and live-dead staining with calcein AM and propidium iodide was performed. In Fig. 5, overlay photographs of encapsulated cells exposed to the acoustics are shown together with a control group of encapsulated cells not exposed to the acoustics. Without the acoustics applied the cells were randomly distributed in the droplets (Fig. 5a), while with the acoustics applied the cells were focused along the centre line in all cases (Fig. 5b–d).

**Figure 6:**
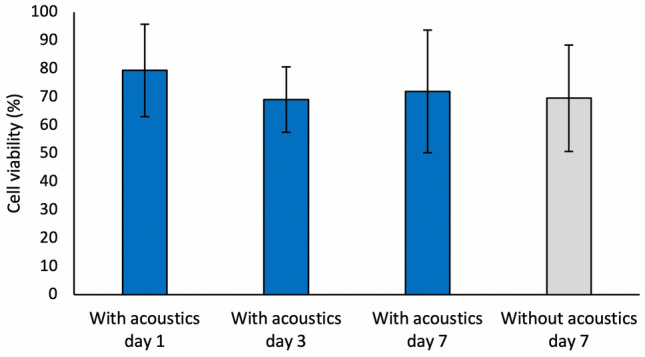
The viability of acoustically focused astrocytes in hydrogel droplets after 1, 3 and 7 days of culture. As a control group, astrocytes encapsulated in hydrogel droplets without acoustic focusing is shown after 7 days of culture.

The cell viability was quantified, and as seen in Fig. 6 the acoustics did not decrease the cell viability. This is in accordance with previous studies that have shown that acoustic manipulation in this frequency and acoustic energy range is not harmful for cells^31^. Regarding the gel composition, it has previously been shown that PEG hydrogels combined with GelMA support cell survival^42^ and this can also be seen from our results.

Our results show that acoustic focusing is a suitable technology for creating hydrogel droplets with controlled cell positioning and maintained high cell viability. Future work involves encapsulating other cell types and investigate different hydrogel materials with different viscosities, stiffness and chemical compositions.

## Conclusion

In this work we have developed a microfluidic system where it is possible to focus beads and cells inside hydrogel droplets on-chip in a direct manner using bulk acoustic standing waves. In the microfluidic chip, droplets composed of a UV-light cross-linkable hydrogel precursor solution with particles were generated. The encapsulated particles were focused to the centre line of the droplets (i.e. the pressure nodal line) by the acoustics, followed by UV cross-linking of the droplets which froze the encapsulated particles at their positions. The system is flexible, and we show that it can be used to focus both polystyrene beads and astrocyte cells. We believe that the presented technology will be a valuable tool for the generation of advanced biological hydrogel structures where the spatial position of encapsulated beads or cells needs to be precisely controlled.

## Material and methods

### Microfluidic chip fabrication

The microfluidic channels were etched on a 500 µm thin silicon wafer using deep reactive ion etching according to a standard protocol^38^. Both the droplet generator channels and the acoustic focusing channel were 380 µm by 100 µm (width × height). The width was set to correspond to half wavelength lateral resonance at approximately 2 MHz in the channel. The inlet and outlet holes were manually drilled through the silicon wafer. The microfluidic channels were sealed by anodic bonding of a 700 µm thin Borofloat-33 glass wafer and the silicon-glass wafer was diced into individual chips. A piezoelectric transducer having a fundamental resonance at 2 MHz (1 mm thickness, APC-840, Americanpiezo) was glued on the silicon side of the chip using cyanoacrylate glue (Loctite 420, Henkel). As fluid connectors, 1 cm long pieces of silicone tubing (228-0701, VWR) were glued using silicone adhesive (Elastosil A07, Wacker). Prior to the experiments the channels were flushed with Repel-Silane (2% solution of dimethyldichlorosilane dissolved in octamethylcyclotetrasiloxane, Pharmacia Biotech) to make the channels hydrophobic followed by rinsing the channels with heavy mineral oil (Sigma Aldrich) to remove unbound Repel-Silane.

### Experimental setup

**Figure 7:**
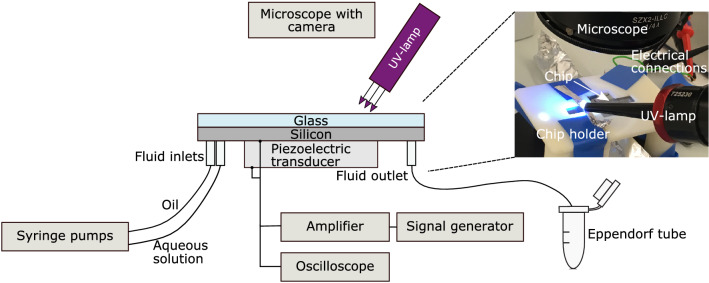
Illustration of the experimental setup. The inset shows the position of the UV-lamp and the chip.

An overview of the experimental setup is shown in Fig. 7. The microfluidic chip was mounted in a 3D printed chip holder and observed in a stereomicroscope (SZX10, Olympus) equipped with a camera (E3CMOS with 6.3 MP sensor, Touptek). A stereomicroscope was chosen as it makes it easy to fit a handheld UV-lamp (APM UV-Cure, APM-Technica) from above. The wavelength of the light was 365 nm. The position of the UV-lamp was carefully adjusted so that the light beam was directed towards the end of the acoustic focusing channel. To prevent unwanted cross-linking, parts of the channel were masked using dark nail polish and aluminium foil. The piezoelectric transducer was actuated by a sine signal from a signal generator (AFG1022, Tektronix) after amplification of the signal by an amplifier (XPA125B, Xiegu). The signal over the piezoelectric transducer was monitored with an oscilloscope (TBS1052B, Tektronix). The inlet fluid flows (one for the hydrogel precursor solution and two for the oil) were controlled by three syringe pumps (neMESYS, Cetoni) mounted with plastic syringes (BD Plastics) and the outlet was connected via a 10 cm long piece of tubing to an Eppendorf tube to collect the hydrogel droplets.

### Cells

Mouse brain astrocyte cells (C8-D1A, ATCC) were cultured in Dulbecco’s Modified Eagle’s Medium (DMEM) high glucose medium (Sigma Aldrich) supplemented with 10% fetal bovine serum HyClone (Fisher Scientific) and 1% Penicillin Streptomycin mixture (Fisher Scientific). Cells were incubated at 37 ºC in 5% CO_2_ to reach 70% confluency. After detaching cells from the cell culture flask by 3 min incubation with TrypLE Express Enzyme (Thermo Fisher), 5 × 10^6^ cells/mL passage 29, were centrifuged, re-suspended in DMEM high glucose and mixed with the hydrogel precursor solution in PBS.

### Methacrylated gelatine (GelMA) synthesis

GelMA synthesis was adapted from Lee et al.^43^. Briefly, 4% (w/v) bovine gelatine (Bloom 225, Sigma Aldrich) was dissolved in deionised water (DI water) and heated to 37 °C. pH was adjusted with NaOH solution to 9 prior and in between every methacrylic anhydride aliquot addition (1.9 mmol per g gelatine in 10 aliquots). Subsequent to methacrylic anhydride addition, the pH was adjusted every 30 min until it remained constant and then stirred vigorously over night at 37 °C. The crude product was purified by dialysis in a dialysis membrane (MWCO 12,000–14,000 g/mol) against DI water. The water was changed twice a day for 4 days. The purified product was freeze dried and stored at -20 °C until use. Degree of functionalisation was determined by ^1^H NMR and calculated to be 54%.

### Experimental procedure

To prepare the hydrogel precursor solution, 3% (w/v) 4arm-PEG10K-Acrylate (Sigma Aldrich) and 0.1% (w/v) lithium phenyl-2,4,6-trimethylbenzoylphosphinate (LAP, Sigma Aldrich) were mixed in phosphate-buffered saline (DPBS, Thermo Fisher 1:9 diluted with DI water). In the bead focusing experiments 10% green fluorescent polystyrene beads (10 µm, Thermo Fisher) and 10% red fluorescent polystyrene beads (0.5 µm, Thermo Fisher) were added to the hydrogel precursor solution. In the cell focusing experiments, 5 × 10^6^ astrocytes/mL were added to the hydrogel precursor solution. In the cell focusing experiments, GelMA was added to the final concentrations (w/v) of PEG, GelMA and LAP of 3%, 1% and 0.1%, respectively. To be able to visualise dead cells, no red fluorescent beads were added to the hydrogel precursor solution. The continuous phase was heavy mineral oil (Sigma Aldrich) with 1% Span80 surfactant (Sigma Aldrich). The hydrogel precursor solution was injected at a flow rate of 1.0 µL/min and the oil was injected at a total flow rate of 1.5 µL/min, if not stated otherwise.

Droplets were generated, passed along the acoustic focusing channel, exposed to the UV-light and collected in an Eppendorf tube. The piezoelectric transducer was actuated to generate a half wavelength acoustic standing wave field in the channel. The frequency was selected based on strong focusing seen in the channel at that frequency. For the bead focusing experiments the frequency was 1.88–1.89 MHz and for the cell focusing experiments the frequency was 1.87 MHz. The amplitude of the signal over the piezoelectric transducer was 30 V_pp_ in all experiments except for the experiment with different droplet lengths where the voltage was 18 V_pp_. Control experiments without the acoustics applied were performed. The collected droplets were washed by adding PBS to the Eppendorf tube and letting the phases separate. The hydrogel droplets were placed on a microscope slide, and observed in a microscope (IX73, Olympus) equipped with a camera (Orca-Flash 4.0 LT digital CMOS camera).

### Characterisation of the acoustic focusing

The degree of focusing of the beads was determined from fluorescent images using ImageJ software. First the area of each droplet was manually marked and the Feret’s angle was calculated. Then the droplet was rotated and aligned along the x-axis in the image. The y-coordinate of the Feret’s diameter (FeretY) was calculated, and the minimum Feret’s diameter (MinFeret) was calculated and used as an estimation of the droplet width. A ROI was defined as (FeretY ± 0.1*MinFeret). The number of beads inside and outside the ROI was manually counted for 20 droplets without and with the acoustics applied.

### Characterisation of cell viability

Cell viability was assessed by performing a live/dead stain after culture of cells in the cross-linked hydrogel droplets at 37 ºC in 5% CO_2_. Cell viability was measured after 1 day, 3 days and 7 days of culture for cells exposed to the acoustics. As a control group cell viability was measured after 7 days of culture for cells not exposed to the acoustics. The hydrogel droplets were washed twice with MEM transparent media (Gibco MEM, no glutamine, no phenol red, Fisher Scientific) by gently pipetting up and down and centrifuging at 500 rpm. Staining was performed by incubating the cell-laden hydrogel droplets for 15 min with 2 µM calcein AM (Fisher Scientific) and 1 µM propidium iodide (Fisher Scientific) in MEM. Cells were washed with MEM once and visualised immediately after staining. Cell viability was measured by manually counting the number of living and dead cells in the droplets. For each experimental group 197–214 cells were counted.

### Viscosity measurements

Frequency dependent viscosity was measured with a TA 2000 rheometer using a 40 mm 2° steel cone geometry at 21 °C. The flow sweep measurement was performed from 0.01 to 10 Hz with a gap distance of 66 μm. Values are reported for 5.5 Hz shear rate.

### Rheology measurements

Mechanical properties of the hydrogels were measured on hydrogel disks (8 mm in diameter and 1.5 mm in height) with an 8 mm stainless steel parallel plate geometry using a Discovery Hybrid Rheometer 2 (TA Instruments). Measurements were performed at 37 °C and an axial force of 30 mN. Storage modulus is reported from frequency sweep experiments (0.1 − 8 Hz at oscillation strain of 0.267%) as average value of triplicate measurements at 1 Hz.
