# Author Correction: Acoustic focusing of beads and cells in hydrogel droplets

**DOI:** 10.1038/s41598-021-96894-4

**Published:** 2021-08-26

**Authors:** Anna Fornell, Hannah Pohlit, Qian Shi, Maria Tenje

**Affiliations:** 1 Department of Materials Science and Engineering, Science for Life Laboratory, Uppsala University, 75121 Uppsala, Sweden; 2 MAXIV Laboratory, Lund University, 22484 Lund, Sweden

Correction to: *Scientific Reports* 10.1038/s41598-021-86985-7, published online 05 April 2020

The original version of this Article contained an error in the Funding section.

“Open access funding provided by Uppsala University. This research was funded by the Knut and Alice Wallenberg Foundation (WAF 2016.0112) and the European Research Council (ERC-2017-STG-757444).”

now reads:

“Open access funding has been provided by Uppsala University. This research has received funding from the Knut and Alice Wallenberg Foundation (WAF 2016.0112), the Swedish Research Council (2019-03797) and from the European Research Council (ERC) under the European Union’s Horizon 2020 research and innovation programme (grant agreement No 757444).”

The original Article has been corrected.

